# Evidence for Clinical Skills Training Using Virtual Reality in Nursing and Midwifery Education: Protocol for an Umbrella Review

**DOI:** 10.2196/94779

**Published:** 2026-09-25

**Authors:** Tshepo Kabelo Mohale, Wilma ten Ham- Baloyi, Olivia Baorapetsi Baloyi, Emelda Zandile Gumede

**Affiliations:** 1 Department of Advance Nursing Science, Faculty of Health Sciences, University of Venda, University Road, Thohoyandou, Venda, Limpopo, 0938, South Africa, 27 0159629456 ext 9456; 2 Department of Nursing Science, Nelson Mandela University, Gqeberha, Eastern Cape, South Africa; 3 Department of Nursing, Faculty of Health Sciences, Walter Sisulu University, Mthatha, Eastern Cape, South Africa; 4 Department of Nursing and Public Health, University of KwaZulu-Natal, Durban, KwaZulu-Natal, South Africa

**Keywords:** virtual reality, immersive technology, nursing education, midwifery education, clinical skills, clinical competence, umbrella review, systematic review, A Measurement Tool to Assess Systematic Reviews, version 2, AMSTAR 2, Grading of Recommendations Assessment, Development and Evaluation, GRADE

## Abstract

**Background:**

Virtual reality (VR) is increasingly used in health care training and nursing education to address clinical skills gaps and provide immersive learning experiences. While multiple systematic reviews have synthesized primary research on VR effectiveness, no umbrella review has comprehensively evaluated and integrated findings from existing systematic reviews specifically focused on nursing and midwifery education.

**Objective:**

This umbrella review aims to systematically identify, critically appraise, and synthesize findings from published systematic reviews and meta-analyses examining the use of VR in nursing and midwifery education, with particular emphasis on clinical skills training.

**Methods:**

This protocol will follow the PRISMA-P (Preferred Reporting Items for Systematic Review and Meta-Analysis Protocols) 2015 statement and is registered with PROSPERO (CRD420261303572). Nine databases will be searched, including PubMed, CINAHL, Scopus, Web of Science, ERIC, PsycInfo, Cochrane Library, African Journals Online, and SciELO. Two independent reviewers will conduct screening, data extraction, and methodological appraisal using AMSTAR 2 (A Measurement Tool to Assess Systematic Reviews, version 2). The overlap of primary studies across reviews will be calculated using the corrected covered area (CCA) method, and decision rules for managing overlap will be applied. Narrative synthesis will follow Synthesis Without Meta-Analysis guidance. Certainty of evidence will be assessed using an adapted GRADE (Grading of Recommendations Assessment, Development and Evaluation) approach with a prespecified framework for downgrading based on AMSTAR 2 ratings, overlap, heterogeneity, imprecision, and indirectness.

**Results:**

The review search and screening commenced in July 2026 and are ongoing as of August 2026. Full-text screening and data extraction are scheduled for September to October 2026, followed by methodological appraisal, overlap assessment, and evidence synthesis from October to December 2026. Manuscript preparation is planned for November to December 2026, with the review findings expected to be submitted for publication in early 2027.

**Conclusions:**

This umbrella review will provide the first comprehensive, high-level synthesis of evidence on the use of VR in nursing and midwifery clinical training. By systematically appraising and grading the certainty of existing systematic reviews, it will identify the most robust findings while exposing critical evidence gaps. The resulting evidence gap map and summary of findings will offer clinicians, policymakers, and researchers a clear roadmap for evidence-based decision-making and prioritization of future primary research.

## Introduction

### Background

The global health care environment is transforming rapidly in response to technological advances, evolving population health needs, and increasingly complex patient care expectations. Nurses and midwives constitute the majority of the health care workforce globally and are central to achieving universal health coverage and the Sustainable Development Goals [1,2]. Their education and ongoing clinical training are therefore critical to improving health outcomes, particularly in resource-constrained settings such as South Africa and other African countries.

Traditional teaching methodologies, including lectures and observational clinical placements, often fall short of equipping students with the hands-on, decision-making, and critical-thinking skills required for contemporary practice [3]. These conventional modalities may not provide adequate exposure to rare but critical clinical events, nor consistently promote learner engagement or the safe exploration of clinical judgment [4]. Growing restrictions on clinical placement availability, patient safety concerns, and educator workload further limit traditional education approaches [5].

Virtual reality (VR) has emerged as a promising educational innovation that addresses these challenges. VR refers to computer-generated environments simulating real-life clinical scenarios, allowing students to practice skills and make decisions in risk-free, engaging, and repeatable settings [6]. Research indicates that VR can enhance psychomotor skills, clinical reasoning, and student satisfaction compared to traditional methods [7]. VR-based birthing simulations have demonstrated improved confidence and decision-making among midwifery students [8], while usability studies report increased realism and engagement [9].

The proliferation of primary studies on VR in nursing and midwifery education has led to numerous systematic reviews and meta-analyses synthesizing this evidence. Recent meta-analyses have demonstrated that VR significantly improves knowledge acquisition, skill performance, and problem-solving abilities in nursing education, with effects varying by immersion level and learner population [10,11]. Chen et al [12] conducted a meta-analysis demonstrating the effectiveness of VR for knowledge acquisition and skill performance. Liu et al [4] provided an updated systematic review and meta-analysis confirming positive effects on clinical competence. Kiegaldie and Shaw [13] examined the feasibility and effectiveness of VR across nursing contexts. In midwifery education specifically, emerging evidence suggests VR enhances student engagement and learning outcomes in complex clinical scenarios such as anatomy instruction and childbirth training [14]. Most recently, Tene et al [15] published an umbrella review of VR and augmented reality across all medical education, noting that nursing and midwifery-specific syntheses remain limited.

The growing number of systematic reviews on virtual and immersive technologies in nursing and midwifery education signals a field that is both dynamic and rapidly evolving. A quick scan of PROSPERO records illustrates this diversity: from early effectiveness reviews such as the review conducted in 2018 on VR in nursing education [16], to more recent syntheses comparing desktop versus immersive VR [17], focused explorations of pediatric and psychiatric applications [18], broader reviews of simulation, and even studies incorporating AI [19].

This richness, however, comes with fragmentation. Each review tends to pursue its own narrow question, whether about student experience, obstetric training, or specific clinical domains, using different methodologies, populations, and definitions of technology. While findings often overlap, they remain siloed. As a result, we lack a consolidated understanding of how evidence aligns across clinical areas, whether certain immersive tools consistently outperform others, and how robust or trustworthy the existing reviews truly are.

An umbrella review provides the opportunity to step back and integrate these different threads. By systematically examining where findings converge, where they diverge, and where evidence is genuinely strong, we can offer a more coherent picture of immersive VR in clinical skills education. Importantly, this is not just an academic exercise. Our intention is to produce a synthesis that is directly useful to educators and decision-makers. Beyond mapping effectiveness, we will pay close attention to implementation—how these technologies can be adopted, adapted, and optimized in nursing education settings.

The ultimate goal is to move beyond isolated findings and provide a clear, evidence-informed guide for the next stage of practice and policy. In doing so, this umbrella review seeks to bridge the gap between research and application, ensuring that the promise of immersive technologies translates into meaningful improvements in nursing and midwifery education.

An umbrella review, a systematic review of existing systematic reviews and meta-analyses, is therefore timely and necessary. Umbrella reviews occupy the highest level of the evidence hierarchy, providing comprehensive overviews for decision-makers while identifying inconsistencies, gaps, and methodological limitations across existing syntheses [20,21]. This approach will consolidate evidence on VR effectiveness specifically for nursing and midwifery education, inform curriculum development and technology integration, and guide future research priorities.

### Objectives

This umbrella review aims to systematically identify, appraise, and synthesize findings from published systematic reviews and meta-analyses on the use of VR in nursing and midwifery education. The primary objectives are to identify and describe existing systematic reviews and meta-analyses evaluating VR interventions in nursing and midwifery education; to appraise the methodological quality of included reviews using the AMSTAR 2 instrument; to quantify primary study overlap using the corrected covered area (CCA); to synthesize findings regarding VR effectiveness for clinical skills acquisition, learner engagement, and higher-order thinking skills; and to assess the certainty of the evidence using an adapted GRADE approach.

## Methods

### Overview

This umbrella review protocol follows the PRISMA-P (Preferred Reporting Items for Systematic Reviews and Meta-Analysis Protocols) guidelines [22]. The completed review will be reported in accordance with the PRISMA (Preferred Reporting Items for Systematic Reviews and Meta-Analyses) 2020 statement (Checklist 1). The review is registered with PROSPERO (CRD420261303572). The research questions guiding this review are as follows: (1) what systematic reviews and meta-analyses have been published on VR use in nursing and midwifery education, and what are their characteristics; (2) what is the methodological quality of existing systematic reviews on VR in nursing and midwifery education, as assessed by AMSTAR 2 (A Measurement Tool to Assess Systematic Reviews, version 2); (3) to what extent do primary studies overlap across included reviews, and how might this affect confidence in synthesized findings; (4) what does the synthesized evidence indicate about VR effectiveness for clinical skills acquisition, learner engagement, and higher-order thinking skills in nursing and midwifery students; and (5) what gaps exist in the current evidence base, and what priorities for future research can be identified?

### Eligibility Criteria

The eligibility criteria for this umbrella review are organized according to the PICOS (population, intervention, comparators, outcomes, and study design) framework with additional contextual and publication parameters. Table 1 presents the complete inclusion and exclusion criteria. Table 2 presents the eligible technologies.

Regarding population scope, reviews that include mixed health care professional populations (eg, physicians, allied health staff, and nurses and midwives) are considered eligible only if nursing and/or midwifery outcome data are reported separately and extractable. This criterion ensures that the synthesis remains specific to the nursing and midwifery workforce and prevents dilution of findings by nonrepresentative pooled estimates. Reviews in which nursing and midwifery participants constitute the majority (>50%) of the included population, but where separate extractable data are not provided, are excluded to avoid overgeneralization. This targeted approach ensures that our conclusions are directly applicable to the research question and are not skewed by populations that are not central to the review’s focus.

**Table 1.** Inclusion and exclusion criteria.

	Inclusion criteria	Exclusion criteria
Population	Studies focusing on nursing students, midwifery students, nursing educators, midwifery educators, or registered nurses and midwives engaged in educational or training activities. Reviews must specifically address nursing and/or midwifery populations, either exclusively or as a clearly identifiable subgroup with separate analyses.	Reviews focusing on other health care professions (medicine, pharmacy, and allied health) without separate nursing or midwifery analyses. Reviews of interprofessional education where nursing or midwifery data cannot be disaggregated. Reviews with mixed health care populations are included only if nursing or midwifery data are separately extractable.
Intervention	Interventions using VR^a^ technology for educational or training purposes. VR refers to computer-generated, 3D environments that users can interact with, providing immersion and presence [23]. VR is classified into immersive VR (fully immersive systems using head-mounted displays), semi-immersive VR (large-screen projections and cave automatic virtual environments), and nonimmersive VR (desktop-based 3D virtual environments).	Screen-based videos, standard e-learning modules, serious games without a VR environment, manikin-only simulation, augmented reality, and mixed reality. For desktop VR and virtual simulation studies to be included, the technology must meet both the following criteria: 3D rendering with spatial depth and perspective manipulation, and active interactivity where users make meaningful decisions or perform actions that alter the environment state (not merely clicking through linear content).
Comparators	Any comparator, including traditional teaching methods, lectures, clinical placements, other simulation modalities, or no intervention. Reviews without comparators (eg, descriptive studies of VR implementation) are included if they meet other criteria, with this noted in synthesis.	No specific exclusions apply to comparators.
Outcomes	Primary outcomes include clinical skills acquisition (objective structured clinical examinations, skills checklists, and procedural competence assessments), learner engagement (self-report measures, observational measures, and completion rates), and higher-order thinking skills (critical thinking, clinical reasoning, decision-making, and problem-solving). Secondary outcomes include knowledge acquisition, confidence and self-efficacy, satisfaction with learning experience, skill retention, feasibility and implementation outcomes (cost, time, and technical requirements), and educator and student perceptions.	Reviews that do not report any of the specified primary or secondary outcomes.
Study design	Published systematic reviews and meta-analyses that report clear, reproducible search strategies and risk-of-bias assessments..	Unpublished completed reviews, registered but unpublished reviews, and gray literature are excluded from the evidence synthesis, quantitative overlap calculations, and certainty grading. Narrative or scoping reviews, conference abstracts, editorials, and opinion pieces are also excluded.
Context	Studies on the use of VR in nursing and midwifery within the clinical or educational training context.	Studies conducted exclusively in noneducational or nonclinical settings.
Language	Studies that are published in English.	Non-English language publications.
Date restrictions	Publications from January 2020 to June 2026.	Publications before January 2020 or after June 2026.

a VR: virtual reality.

**Table 2.** Eligible technologies.

Technology	Included	Excluded
Head-mounted VR^a^ simulation	✓	
Oculus or Vive training systems	✓	
Desktop 3D virtual ward simulation	✓	
Projection-based VR environments	✓	
2D screen-based videos		✓
Standard e-learning modules		✓
Serious games without a VR environment		✓
Manikin-only simulation		✓
Augmented reality		✓
Mixed reality		✓

a VR: virtual reality.

### Information Sources

The following electronic databases will be searched: PubMed and MEDLINE (January 2020 to June 2026), CINAHL (January 2020 to June 2026), Scopus (January 2020 to June 2026), Web of Science Core Collection (January 2020 to June 2026), ERIC (January 2020 to June 2026), PsycInfo (January 2020 to June 2026), Cochrane Library (including Cochrane Database of Systematic Reviews; January 2020 to June 2026), African Journals Online (January 2020 to June 2026) for African-published reviews, and SciELO (January 2020 to June 2026) for Latin American and African content. No unpublished, registered-only, or gray literature sources will be included in the evidence synthesis, overlap calculations, or GRADE (Grading of Recommendations Assessment, Development and Evaluation) assessments. Gray literature searches will be conducted for informational purposes only and will not be assessed for eligibility.

### Search Strategy

The search strategy will be developed in consultation with an experienced health sciences librarian. A 3-step process will be followed. Step 1 involves an initial limited search of PubMed and CINAHL to identify relevant keywords and index terms from retrieved articles. Step 2 involves the development of a comprehensive search strategy using identified keywords and subject headings, adapted for each database. Step 3 involves the examination of reference lists of included reviews to identify additional studies. Table 3 shows the preliminary search strategy (PubMed format).

Detailed search strategies for each database will be included as appendices in the final published protocol and review.

**Table 3.** Preliminary search strategy^a^.

Concept	Search terms
Population	(“nursing”[Mesh] OR “nursing education”[Mesh] OR “nursing students”[Mesh] OR “midwifery”[Mesh] OR “nurse midwives”[Mesh] OR “nursing”[tiab] OR “nurses”[tiab] OR “midwifery”[tiab] OR “midwives”[tiab] OR “nurse education”[tiab] OR “midwifery education”[tiab])
Intervention	(“virtual reality”[Mesh] OR “virtual reality”[tiab] OR “VR”[tiab] OR “immersive technology”[tiab] OR “immersive simulation”[tiab] OR “computer simulation”[Mesh] OR “simulation training”[Mesh:nocxp] OR “virtual simulation”[tiab])
Study type	(“systematic review”[pt] OR “systematic review”[tiab] OR “meta-analysis”[pt] OR “meta-analysis”[tiab] OR “meta analyses”[tiab] OR “systematic literature review”[tiab] OR “systematic review”[All Fields])
Date	AND (“2020/01/01”[PDAT]: “2026/02/13”[PDAT])

a Combined: #1 AND #2 AND #3 AND #4.

### Study Selection Process

Selection will follow a 2-stage process conducted by 2 reviewers (TKM and OBB) who will independently screen titles and abstracts and full texts. Disagreements will be resolved through adjudication by a third reviewer (WtH-B). Citation management will be performed using EndNote (version 21; Clarivate), and screening will be facilitated using Covidence to ensure efficient and transparent tracking of decisions. A PRISMA 2020 flow diagram will be used to document the selection process. Specifically, this umbrella review will use the PRISMA 2020 flow diagram for updated systematic reviews that incorporate searches from databases and registers only, as no other sources will be included in the evidence synthesis [24,25].

### Data Extraction

A standardized data extraction form will be developed and piloted across 3 included reviews by 2 reviewers (TKM and OBB) independently. Refinements will be made to ensure consistency and comprehensiveness. Table 4 shows the data to be extracted.

Data extraction will be performed by 2 reviewers (TKM and OBB) independently and verified by a third reviewer (WtH-B) for accuracy. Disagreements will be resolved through discussion.

**Table 4.** Data extraction.

Category	Specific items
Review characteristics	Authors, year, journal, country of corresponding author, funding sources, and conflicts of interest
Methods	Objectives, search dates, databases searched, inclusion and exclusion criteria, quality appraisal tool used, synthesis method (narrative, meta-analysis, or both), and software used
Population details	Number of included studies, total participants, populations included (nursing students, midwifery students, educators, and qualified professionals), settings (academic and clinical), and countries represented
Intervention details	Type of VR (immersive, semi-immersive, or nonimmersive), VR content and scenarios, duration and frequency, and setting (simulation laboratory, classroom, or home)
Comparator details	Types of comparators used
Outcome details	Outcomes measured, measurement tools, main findings (direction and magnitude of effect), and heterogeneity assessments (if meta-analysis)
Quality appraisal	Risk of bias and quality assessment results (summary and per study if reported)
Review conclusion	Authors’ main conclusions, limitations acknowledged, and implications for practice and research
Primary study	List of primary studies included in the review

### Citation Management and Screening

All records will be exported into EndNote for reference management and duplicate removal. Following deduplication, citations will be imported into Covidence systematic review software (Veritas Health Innovation) to facilitate title and abstract screening, full-text review, reviewer conflict resolution, and audit trail documentation. Data extraction forms will be piloted within Covidence and exported for analysis. The use of dedicated review software will enhance transparency, reproducibility, and management of reviewer decisions throughout the review process.

### Methodological Quality Appraisal

Quality of included reviews will be assessed using AMSTAR 2, a critical appraisal tool for systematic reviews of health care interventions that include randomized and/or nonrandomized studies [24]. AMSTAR 2 evaluates 16 items across key methodological domains, including the definition of research questions and inclusion criteria, protocol registration, justification for included study designs, adequacy of the literature search strategy, duplicate study selection and data extraction, provision of a list of excluded studies with justifications, adequate description of included studies, appropriate assessment of risk of bias, reporting of funding sources, appropriate use of meta-analytical methods, assessment of the impact of risk of bias on results, interpretation of risk of bias in discussing results, explanation of heterogeneity, assessment of potential publication bias, and reporting of conflicts of interest [26,27]. Reviews will be rated as having high, moderate, low, or critically low confidence based on critical domain assessments.

### Assessment of Primary Study Overlap

#### Overview

Overlap of primary studies across included systematic reviews is a key methodological consideration in umbrella reviews [27]. Overlap will be evaluated using citation matrices and CCA calculations, with guidance from recent methodological recommendations for pairwise overlap assessment in umbrella reviews [28,29]. Table 5 below presents the interpretation of overlap among included systematic reviews.

**Table 5.** Interpretation of overlap among included systematic reviews^a^.

Corrected covered area (%)	Level of overlap
0‐5	Slight
6‐10	Moderate
11‐15	High
>15	Very high

a Where substantial overlap (CCA >10%) is identified, findings will be interpreted cautiously

#### Management of Overlap in Synthesis

When CCA indicates moderate (6%-10%) or high overlap (11%-15 %), the following approaches will be applied. The highest-priority review (based on Table 6 criteria) will be used as the primary source for each outcome domain where multiple reviews cover the same evidence. Lower-priority reviews covering the same primary studies will be used to verify consistency of findings. If findings are consistent across reviews, confidence in the conclusion increases. If findings diverge despite overlapping evidence, methodological differences will be explored as a potential explanation. When overlap is high, findings will be presented with a clear statement that “X reviews report on overlapping bodies of evidence, with Y primary studies appearing in multiple syntheses. The following conclusions are based primarily on the highest-quality/most recent synthesis [citation], with corroboration from other reviews noted where present.” A sensitivity analysis will be conducted excluding reviews with CCA >15% (very high overlap) to assess whether conclusions change when only reviews with unique primary study pools are included. When prioritization criteria conflict (eg, a recent review has lower quality than an older review), the decision will be documented with explicit rationale. In such cases, both reviews will be presented with a transparent discussion of trade-offs. Reviews with CCA>15% will not be excluded but will be clearly identified and given reduced interpretive weight. This approach follows Cochrane guidance on managing overlap in overviews of reviews [30].

**Table 6.** Prioritization hierarchy for reviews covering the same body of evidence^a^.

Priority level	Criterion
1	Highest methodological quality based on AMSTAR 2^b^
2	Most recent search date
3	Most comprehensive review (the largest number of included primary studies)
4	Availability of meta-analytic estimates

a Lower-quality or superseded reviews will not be excluded; however, they will be clearly identified during synthesis and given less weight when drawing conclusions.

b AMSTAR 2: A Measurement Tool to Assess Systematic Reviews, version 2.

### Data Synthesis

#### Synthesis Approach

Given the expected heterogeneity across reviews in populations, interventions, comparators, outcomes, and methods, a narrative synthesis will be the primary approach, following Synthesis Without Meta-Analysis guidelines [31]. If appropriate, pooled meta-analytic estimates will be synthesized quantitatively.

#### Assessment of Certainty in Findings

The overall certainty of evidence for primary outcomes will be assessed using the GRADE approach, adapted for umbrella reviews [32-34]. This adaptation acknowledges that applying GRADE to overviews of reviews requires specific considerations, including addressing both the primary study risk of bias as assessed by the included reviews and the risk of bias of the systematic reviews themselves, as well as the statistical heterogeneity observed in meta-analyses conducted within the included reviews [34].

### GRADE Framework for Umbrella Review

Following established guidance for umbrella reviews, evidence will start at high certainty when multiple high-quality systematic reviews with consistent findings are available. Evidence will start at moderate when only 1 high-quality review or multiple moderate-quality reviews are available. Table 7 shows the downgrading criteria (adapted from GRADE for overviews).

Total downgrade levels will be summed across applicable factors as per Table 8.

The summary of findings table will report the certainty rating, downgrading reasons, and a brief rationale for each primary outcome.

**Table 7.** Downgrading criteria.

Factor and operational definition	Downgrade levels
Methodological limitations	
AMSTAR 2^a^ rating: critically low	Downgrade 2 levels
AMSTAR 2 rating: low	Downgrade 1 level
AMSTAR 2 rating: moderate	No downgrade
Primary study overlap	
CCA^b^ >15% (very high)	Downgrade 2 levels
CCA 11%‐15% (high)	Downgrade 1 level
CCA 6%‐10% (moderate)	Consider downgrade by 1 level if accompanied by other concerns
Heterogeneity	
*I*^2^>75% or inconsistent direction of effects across reviews	Downgrade 1‐2 levels depending on severity
Imprecision	
Total participants <OIS^c^ or CIs cross the null and include a clinically important effect	Downgrade 1 level
Indirectness	
Population, intervention, or outcome differs substantially from the review question	Downgrade 1‐2 levels
Publication bias	
Evidence of funnel plot asymmetry or small-study effects reported in included reviews	Downgrade 1 level

a AMSTAR 2: A Measurement Tool to Assess Systematic Reviews, version 2.

b CCA: corrected covered area.

c OIS: optimal information size.

**Table 8.** Factors for downgrade levels.

Rule or factor	Details
Aggregation (downgrades)	
Total downgrade levels	Summed across all applicable factors (max total=3 levels)
Maximum downgrade	3 levels (from high down to very low)
Floor or cap	Evidence cannot be downgraded below very low
Same-domain additivity	Multiple factors within the same domain (overlap+heterogeneity) are considered additive
Upgrading factors	
Large effect	Upgrade 1 level if RR >2 or <0.5 (based on consistent evidence)
Dose-response	Upgrade 1 level if a dose-response gradient is present
Confounding	Upgrade 1 level if plausible confounders would reduce the observed effect (rather than increase it)
Certainty ratings	
High	Very confident that the true effect is close to the estimate
Moderate	Moderately confident; the true effect is likely close, but there is a possibility of substantial difference
Low	Limited confidence; the true effect may be substantially different
Very low	Very little confidence in the effect estimate

### Ethical Considerations

This study was approved by the University of KwaZulu-Natal Research Ethics Committee for BioMedical Research (BREC/00008434/2025).

## Results

Protocol development commenced in February 2026. PROSPERO registration was submitted in February 2026. Literature searches are scheduled for July to August 2026, with title and abstract screening expected to occur between August and September 2026. Full-text review and data extraction are planned for September to October 2026. Methodological appraisal, overlap assessment, and evidence synthesis are anticipated between October and December 2026. Manuscript preparation is expected to occur during November to December 2026, with submission of the completed umbrella review anticipated in January 2027. The review is currently in the protocol phase, and no data have yet been extracted.

**Figure 1.**
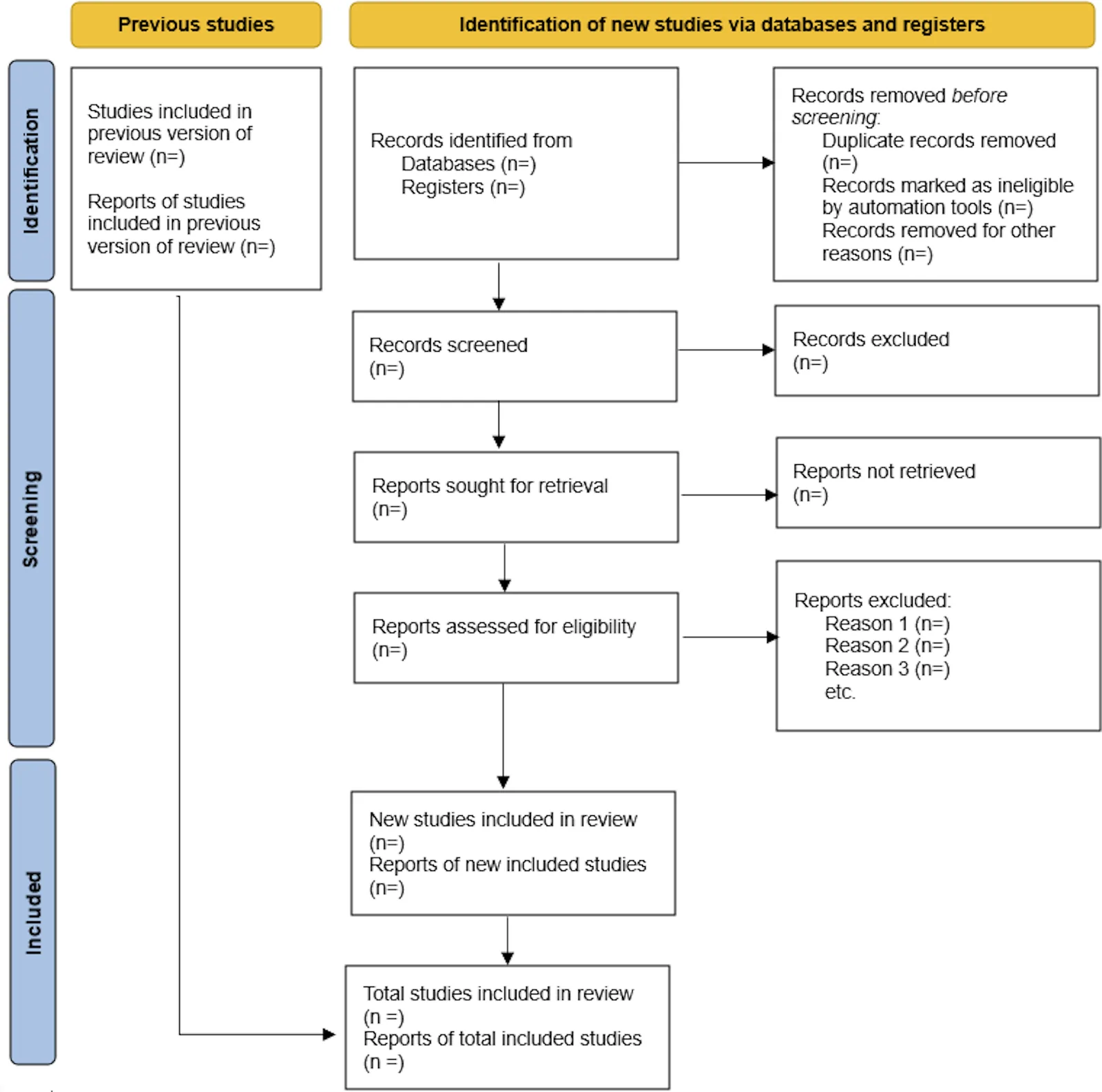
PRISMA 2020 flow diagram for updated systematic review selection (to be completed upon finalization of the review).

The PRISMA 2020 flow diagram will show the number of records identified, screened, and included or excluded. This umbrella review will use the PRISMA 2020 flow diagram designed for updated systematic reviews that incorporate searches from databases and registers [24,25]. The following outputs will be produced: AMSTAR 2 quality tables summarizing the methodological quality ratings of all included reviews, CCA matrices detailing the overlap of primary studies across reviews, outcome synthesis tables narratively summarizing the findings of included reviews for each primary and secondary outcome, GRADE summary of findings tables presenting the certainty of evidence for each primary outcome, and an evidence gap map visually identifying areas with sufficient evidence and areas with insufficient or inconsistent evidence. Figure 1 below presents the PRISMA flow diagram for updated systematic review selection.

## Discussion

### Principal Findings

This umbrella review is expected to provide the first comprehensive synthesis of review-level evidence examining VR interventions specifically within nursing and midwifery education, consistent with recent primary systematic reviews [10,11]. On the basis of findings reported across existing systematic reviews and meta-analyses, it is anticipated that VR-based educational interventions may demonstrate benefits for clinical skills acquisition, learner engagement, knowledge development, confidence, and clinical decision-making. However, the magnitude and consistency of these effects may vary according to immersion level, learner population, educational context, and methodological quality of the evidence.

### Comparison With Prior Work

Previous reviews have evaluated immersive technologies across broader health care and medical education disciplines [15]. However, no umbrella review has focused exclusively on nursing and midwifery education. This review therefore extends previous work by providing profession-specific evidence and evaluating the methodological quality and overlap of existing reviews.

### Strengths and Limitations

A key strength of this review is the use of rigorous umbrella review methodology, including AMSTAR 2 appraisal, overlap assessment using CCA calculations, adherence to PRISMA guidance, and certainty assessment using an adapted GRADE framework. Inclusion of nursing- and midwifery-specific evidence may enhance the relevance of findings for curriculum developers and educators. The protocol is prospectively registered, enhancing transparency.

Potential limitations include dependence on the quality of available systematic reviews, variability in VR definitions across studies, language restrictions to English publications, and possible overlap of primary studies.

### Implications for Practice and Research

Findings may inform evidence-based decisions regarding the integration of immersive technologies into nursing and midwifery curricula, simulation laboratories, continuing professional development programs, and resource allocation decisions. Evidence gaps identified during the review may help prioritize future primary studies and systematic reviews.

### Dissemination Plan

Findings will be disseminated through publication in a peer-reviewed journal, conference presentations, institutional research seminars, and stakeholder engagement activities involving nursing and midwifery educators. A summary of findings will also be shared with curriculum committees and professional education networks to support evidence-informed implementation of VR technologies.

## Supplementary material

10.2196/94779 Checklist 1 PRISMA-P checklist.

## Abbreviations

AMSTAR 2: A Measurement Tool to Assess Systematic Reviews, version 2
CCA: corrected covered area
GRADE: Grading of Recommendations Assessment, Development and Evaluation
PRISMA: Preferred Reporting Items for Systematic Review and Meta-Analyses
PRISMA-P: Preferred Reporting Items for Systematic Reviews and Meta-Analysis Protocols
VR: virtual reality
